# Phosphorylation of Akt and ERK1/2 Is Required for VEGF-A/VEGFR2-Induced Proliferation and Migration of Lymphatic Endothelium

**DOI:** 10.1371/journal.pone.0028947

**Published:** 2011-12-12

**Authors:** Michael T. Dellinger, Rolf A. Brekken

**Affiliations:** 1 Department of Surgery, University of Texas Southwestern Medical Center, Dallas, Texas, United States of America; 2 Hamon Center for Therapeutic Oncology, University of Texas Southwestern Medical Center, Dallas, Texas, United States of America; 3 Department of Pharmacology, University of Texas Southwestern Medical Center, Dallas, Texas, United States of America; Feinberg Cardiovascular Research Institute, Northwestern University, United States of America

## Abstract

There is growing evidence that vascular endothelial growth factor-A (VEGF-A), a ligand of the receptor tyrosine kinases VEGFR1 and VEGFR2, promotes lymphangiogenesis. However, the underlying mechanisms by which VEGF-A induces the growth of lymphatic vessels remain poorly defined. Here we report that VEGFR2, not VEGFR1, is the primary receptor regulating VEGF-A-induced lymphangiogenesis. We show that specific inhibition of VEGF-A/VEGFR2 signaling with the fully human monoclonal antibody r84 significantly inhibits lymphangiogenesis in MDA-MB-231 tumors. *In vitro* experiments with primary human dermal lymphatic endothelial cells (LECs) demonstrate that blocking VEGF-A activation of VEGFR2, not VEGFR1, significantly inhibits VEGF-A-induced proliferation and migration of LECs. We show that VEGF-A stimulation of LECs leads to the phosphorylation of VEGFR2 (Tyr 951, 1054, 1059, 1175, and 1214) which subsequently triggers PKC dependent phosphorylation of ERK1/2 and PI3-K dependent phosphorylation of Akt. Additionally, we demonstrate that inhibitors that suppress the phosphorylation of ERK1/2 and Akt significantly block VEGF-A- induced proliferation and migration of LECs. Together, these results shed light on the mechanisms regulating VEGF-A-induced proliferation and migration of LECs, reveal that VEGFR2 is the primary signaling VEGF-A receptor on lymphatic endothelium, and suggest that therapeutic agents targeting the VEGF-A/VEGFR2 axis could be useful in blocking the pathological formation of lymphatic vessels.

## Introduction

Lymphatic vessels are required for the absorption of intestinal lipids, transport of immune cells, and return of tissue fluid and macromolecules to the blood vascular system [1]. Impaired function of the lymphatic system or an insufficient number of lymphatic vessels can cause the accumulation of fluid and protein in tissues and result in the debilitating disorder lymphedema [2]. Conversely, new lymphatic vessels form in many pathological settings and participate in the progression of several human diseases [2]. These observations have fueled intense research efforts to identify the molecular mechanisms regulating lymphangiogenesis so that therapies can be developed to promote or inhibit this process.

The study of lymphangiogenesis gained momentum following the discovery of the first lymphatic growth factor, vascular endothelial growth factor (VEGF)-C. VEGF-C is indispensable for the proper development of the lymphatic system in several animal models and induces inflammatory and tumor lymphangiogenesis [3], [4], [5], [6], [7], [8]. Although VEGF-C is a robust lymphatic growth factor, it does not act alone. Other members of the VEGF family were recently shown to stimulate the growth of lymphatics [7]. The most prominent member of this family is VEGF-A, a ligand of the receptor tyrosine kinases VEGFR1 and VEGFR2 [9].

VEGF-A is a crucial regulator of embryonic and pathological hemangiogenesis. Inactivation of a single allele of VEGF-A in mice leads to lethality around embryonic day 11.5 because of severe defects in blood vessel development [10], [11]. VEGF-A is also a major regulator of pathological hemangiogenesis that occurs in inflammatory diseases, diabetic retinopathy, and tumors [9]. VEGFR2 is the primary receptor controlling VEGF-A stimulated growth of blood vessels. Mechanistically, VEGF-A/VEGFR2 signaling induces hemangiogenesis by promoting blood endothelial cell (BEC) proliferation, survival, and migration in part through the activation of the mitogen-activated protein kinase/extracellular-signal-regulated kinase-1/2 (ERK1/2) and phosphatidylinositol 3-kinase (PI3-K)/Akt signal transduction pathways [9]. Other additional pathways regulating these cellular processes have been extensively studied and defined in BECs. In contrast, the mechanisms underlying VEGF-A-induced lymphangiogenesis remain poorly defined and controversial.

Interestingly, the *in vivo* response to VEGF-A is strikingly different for lymphatic and blood vessels. Adenoviral mediated delivery of VEGF-A to the ear skin of mice leads to the dramatic enlargement of lymphatic vessels and impairment in lymphatic vessel function [12], [13]. Transgenic overexpression of VEGF-A in the skin of mice also causes lymphatic vessels to preferentially increase in caliber rather than number during settings of inflammation [14], [15]. Conversely, VEGF-A expression in the skin of mice induces sprouting hemangiogenesis resulting in an increase in density of blood vessels [13]. This contrasting effect of VEGF-A on lymphatic and blood vessels raises the possibility that the mechanisms underlying VEGF-A-induced lymphangiogenesis are different than those underlying VEGF-A-induced hemangiogenesis.

It has recently been reported that VEGF-A directly promotes the proliferation and migration of lymphatic endothelial cells (LECs) [16], [17], [18], [19], [20], [21]. Additionally, VEGF-A stimulates the phosphorylation of PLC-γ, Akt and ERK1/2 in LECs [22], [23], [24]. However, the extent to which VEGFR1 and VEGFR2, both of which are expressed by LECs [12], [13], [21], [25], [26], [27], contribute to these events has not been fully delineated. Furthermore, experiments with LECs have not included inhibitors of these molecules/pathways to define the functional significance they serve in promoting VEGF-A-induced processes.

The present study explores the function of VEGF-A/VEGFR2 signaling in promoting the proliferation and migration of LECs. To accomplish this, the novel anti-VEGF-A antibody r84 was used. r84 is a fully human monoclonal antibody that specifically binds VEGF-A and prevents it from activating VEGFR2, but not VEGFR1, in a dose-dependent manner [28]. Here we show for the first time that VEGF-A activation of VEGFR2 directly stimulates LEC proliferation and migration through the PI3-K and ERK1/2 signaling pathways. These experiments shed light on the mechanisms underlying VEGF-A-induced proliferation and migration of LECs and reveal that the circuitry of VEGF-A/VEGFR2 signaling is conserved between LECs and BECs.

## Results

### Blocking VEGF-A activation of VEGFR2 is sufficient to suppress lymphangiogenesis

**Figure 1:**
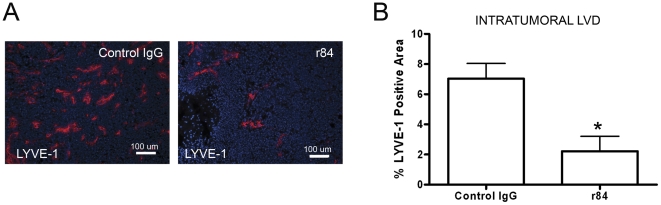
Specific blockade of VEGF-A activation of VEGFR2 suppresses tumor lymphangiogenesis, lymphatic endothelial cell proliferation/viability and migration. **A**: Intratumoral lymphatics were identified by immunofluorescence staining of frozen sections of control IgG and r84 treated MDA-MB-231 tumors for the lymphatic marker LYVE-1 (red). Scale bars = 100 µm. **B**: The entire area of each LYVE-1 stained tumor section was examined at low magnification and the percent of LYVE-1 positive area was determined for each field using NIS-Elements imaging software. Ten fields with the highest LYVE-1 positive percent area were averaged together to yield a final score for each tumor and group means were tested for significance by an unpaired student's t-test. The percent of LYVE-1 positive area of control tumors (7.03±1.013) was significantly greater than r84 treated tumors (2.23±0.986). Asterisk = P = 0.0042.

We previously reported that r84 significantly inhibits hemangiogenesis in MDA-MB-231 tumors [29]. However, we did not examine lymphangiogenesis in this study. To evaluate the effect of r84 on lymphangiogenesis, MDA-MB-231 tumors from our previous study were stained with an antibody against LYVE-1. LYVE-1 positive area was significantly lower in tumors from r84 treated mice (2.23±0.986; n = 5) than in tumors from control IgG treated mice (7.03±1.013; n = 6)(Fig. 1A–C). These data reveal that specifically blocking VEGF-A activation of VEGFR2 with r84 is sufficient to suppress lymphangiogenesis *in vivo*.

**Figure 2:**
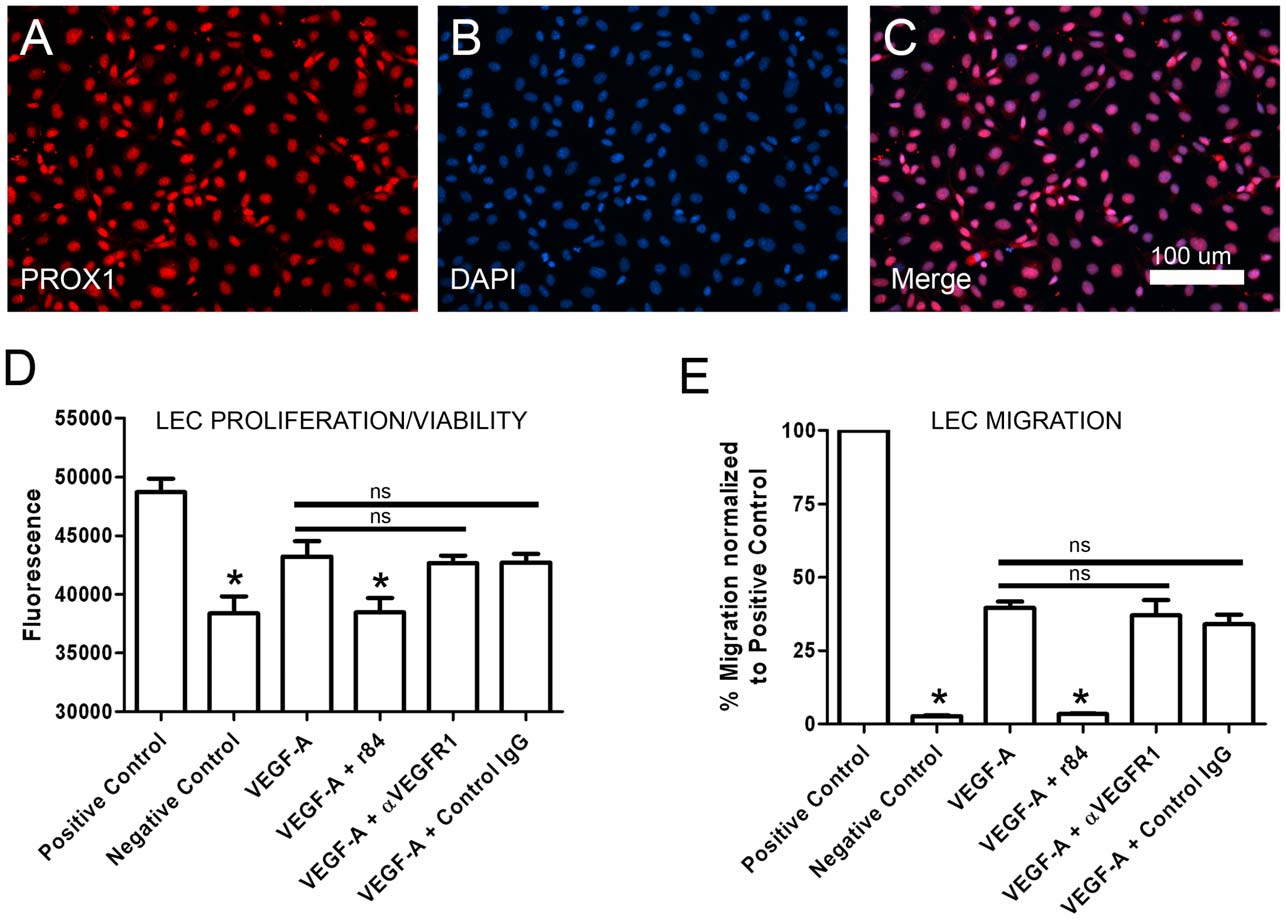
Specific blockade of VEGF-A activation of VEGFR2 suppresses lymphatic endothelial cell proliferation/viability and migration. **A–C**: PROX1 (**A**) is localized in the nucleus (**B**) in primary human LECs (**C**). **D**: Cell viability/proliferation was measured with Cell Titer Blue reagent after culturing LECs for 48 hours in EGM-2MV media (positive control), reduced-serum media (negative control), or with VEGF-A (100 ng/ml) in the presence or absence of r84 (500 molar excess), a functional blocking antibody against VEGFR1 (500 molar excess), or control IgG (500 molar excess). r84 blocked VEGF-A-induced proliferation/viability of LECs whereas the other antibodies had no effect. **E**: LECs were seeded in the upper chamber of a transwell insert and allowed to migrate overnight toward EGM-2MV (positive control), reduced-serum media (negative control), or VEGF-A (100 ng/ml) in the presence or absence of r84 (500 molar excess), a functional blocking antibody against VEGFR1 (500 molar excess), or control IgG (500 molar excess). The number of LECs that migrated to the lower chamber was counted and normalized to the positive control. r84 blocked VEGF-A-induced migration whereas the other antibodies had no effect. For panels C and D, significance tested by ANOVA. Asterisk P<0.05 compared to VEGF-A. ns = not significant compared to VEGF-A.

*In vitro* experiments were then performed with primary human LECs to evaluate the effect of r84 on VEGF-A-induced cellular processes required for lymphangiogenesis. Immunofluorescence staining for the lymphatic marker PROX1 demonstrated that our cultures consisted of a highly pure population of lymphatic endothelium (Fig. 2A–C). Additionally, reverse-transcription PCR confirmed LEC expression of *VEGFR1* and *VEGFR2* (data not shown).

The Cell Titer Blue assay revealed that VEGF-A significantly induced the proliferation of LECs (P<0.05; Fig. 2D). VEGF-A-induced proliferation was not inhibited by a functional blocking antibody against VEGFR1 or by a non-specific control IgG (Fig. 2D). Conversely, blockade of VEGF-A activation of VEGFR2 with r84 significantly (P<0.05) inhibited VEGF-A-induced proliferation of LECs (Fig. 2D).

We next examined the effect of r84 on LEC migration. Transwell migration assays demonstrated that VEGF-A significantly induced the migration of LECs (P<0.05; Fig. 2E). VEGF-A-induced migration was resistant to a functional blocking antibody against VEGFR1 and to a non-specific control IgG, but was completely blocked by r84 (P<0.05; Fig. 2E). These data indicate that VEGF-A activation of VEGFR2, not VEGFR1, directly drives LEC proliferation and migration.

### VEGFR2 is the primary signaling VEGF-A receptor in LECs

**Figure 3:**
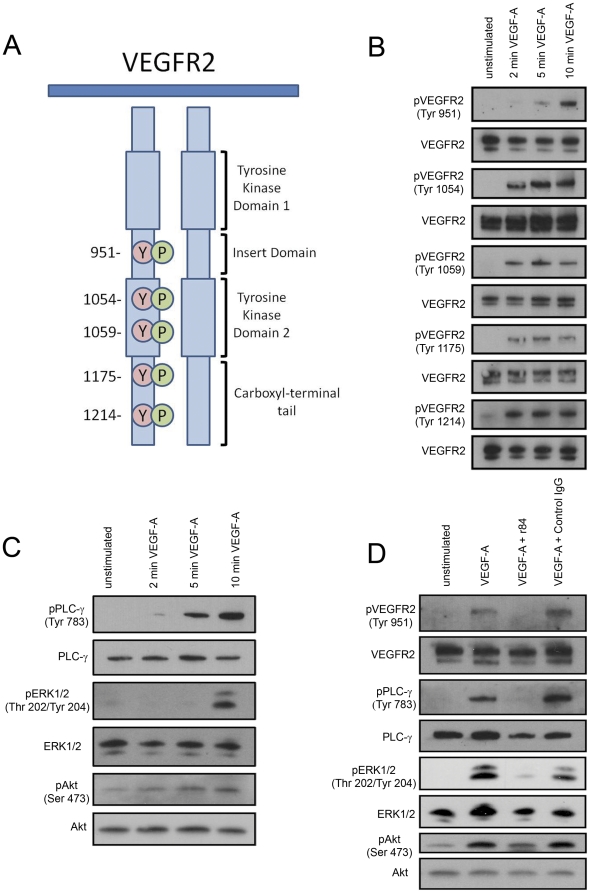
VEGFR2, not VEGFR1, regulates VEGF-A-induced activation of PLC-γ, ERK1/2, and Akt in LECs. **A**: Diagram adapted from [43] depicting phosphorylation sites of the intracellular domain of VEGFR2. **B,C**: Lysates of primary human dermal LECs were made after stimulating LECs with recombinant human VEGF-A (100 ng/ml) for 2, 5, or 10 minutes. The activation of VEGFR2, PLC-γ, ERK1/2 and Akt was detected by Western blotting using phospho-specific antibodies. **D**: Lysates were generated of LECs stimulated with VEGF-A (100 ng/ml, 10 minutes) in the presence or absence of r84 (500 molar excess) or control IgG (500 molar excess). The activation of VEGFR2, PLCγ, ERK1/2 and Akt was detected by Western blotting. r84 suppressed phosphorylation of PLC-γ, ERK1/2, and Akt in LECs.

To achieve a better understanding of how the VEGF-A/VEGFR2 axis promotes LEC proliferation and migration, we analyzed VEGF-A-induced signaling in LECs. VEGF-A triggers the auto-phosphorylation of VEGFR2 on several tyrosine residues that regulate its kinase activity and serve as docking sites for adapter proteins that promote specific signal transduction cascades (Fig. 3A). The phospho-tyrosine profile of VEGFR2 has not been examined previously for VEGF-A-stimulated LECs. Failure in the phosphorylation of one of these key tyrosine residues could dramatically impact kinase activity or downstream signaling in LECs. VEGF-A stimulation of LECs resulted in the phosphorylation of Tyr 951, 1054, 1059, 1175, and 1214 (Fig. 3B). These results indicate that the phospho-tyrosine profile of VEGFR2 is similar between VEGF-A stimulated LECs and BECs.

VEGF-A stimulation of BECs leads to the activation of signaling molecules that serve crucial functions in hemangiogenesis such as PLC-γ, ERK1/2, and Akt. In LECs, VEGF-A stimulated the phosphorylation of PLCγ, ERK1/2, and Akt within 10 minutes (Fig. 3C). Western blot analysis (Fig. 3D) and subsequent quantitation of bands by densitometery revealed that r84 inhibited VEGF-A-induced phosphorylation of VEGFR2, PLCγ, ERK1/2, and Akt by 95%, 64%, 94%, and 67%, respectively.

### VEGF-A promotes PKC dependent phosphorylation of ERK1/2 in LECs

**Figure 4:**
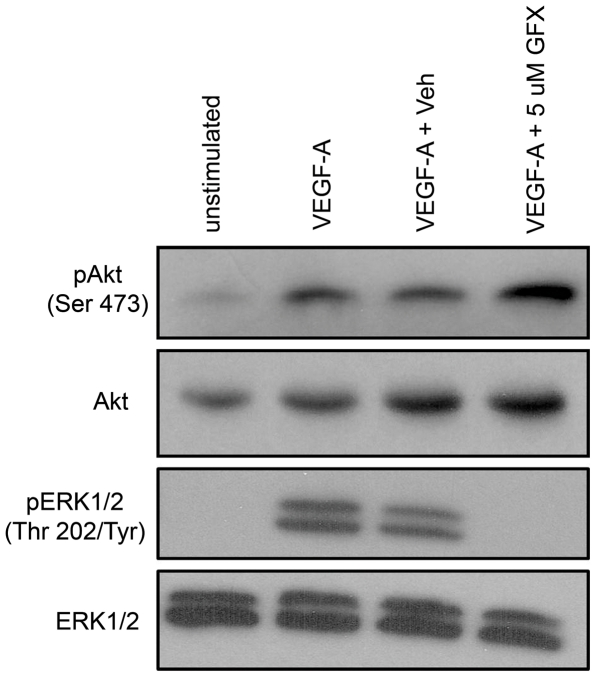
Protein kinase C (PKC) regulates VEGF-A-induced activation of ERK1/2 but not Akt. LECs were maintained in starvation media or treated with DMSO (Veh) or the PKC inhibitor GF10203X (GFX) for one hour prior to stimulation with VEGF-A (100 ng/ml, 10 minutes). Akt and ERK1/2 activation was detected by Western blotting.

Rapid activation of ERK1/2 in VEGF-A-stimulated BECs is controlled primarily by Protein Kinase C (PKC) rather than Ras [30]. To determine whether this circuitry was conserved in lymphatics, primary LECs were stimulated with VEGF-A in the presence of the PKC inhibitor GF109203X (GFX). Western blot analysis showed that ERK1/2 activation was completely blocked by PKC inhibition whereas Akt activation was unaffected (Fig. 4). These data indicate that the topology of the network driving early ERK1/2 activation in BECs and LECs are similar to one another. Furthermore, PKC is not required for Akt phosphorylation.

### Phosphorylation of ERK1/2 and Akt is required for VEGF-A-mediated LEC proliferation and migration

**Figure 5:**
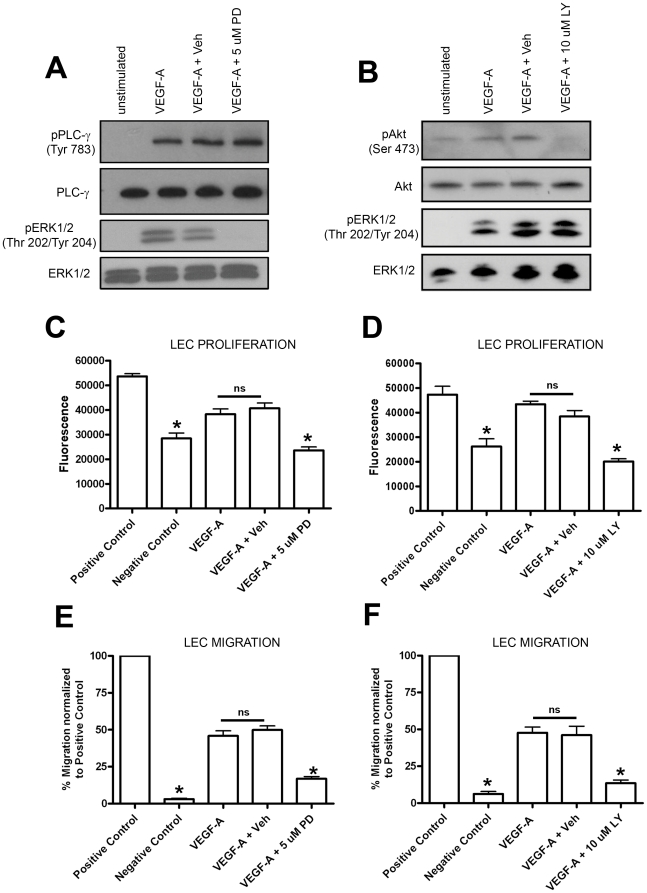
ERK1/2 and Akt regulate VEGF-A-induced proliferation and migration of LECs. **A,B**: Lysates were generated of LECs1) maintained in starvation media, 2) treated with VEGF-A (100 ng/ml, 10 minutes), 3) pretreated with DMSO (Veh) in starvation media for one-hour then stimulated with VEGF-A (100 ng/ml, 10 minutes), or 4) pretreated with the MEK inhibitor PD098059 (PD) or PI3-K inhibitor LY294002 (LY) for one-hour then with stimulated with VEGF-A (100 ng/ml, 10 minutes). PLC-γ, ERK1/2, and Akt activation was detected by Western blotting. **C,D**: Cell viability/proliferation was measured with Cell Titer Blue reagent after culturing LECs for 48 hours in EGM-2MV media (positive control), reduced-serum media (negative control), or with VEGF-A (100 ng/ml) in the presence of DMSO (Veh), PD, or LY. PD and LY blocked VEGF-A-induced proliferation/viability of LECs. **E,F**: LECs were seeded in the upper chamber of a transwell insert and allowed to migrate overnight toward EGM-2MV (positive control), reduced-serum media (negative control), or VEGF-A (100 ng/ml) in the presence or absence of DMSO (Veh), PD, or LY. DMSO, PD, and LY were also included in the upper chamber of the transwell insert. The number of LECs that migrated to the lower chamber were counted and normalized to the positive control. PD and LY inhibited LEC migration toward VEGF-A. For panels B,C, E, and F, significance was tested by ANOVA. Asterisk P<0.05 compared to VEGF-A. ns = not significant compared to VEGF-A.

To determine the function ERK1/2 and Akt phosphorylation serves in VEGF-A-induced processes, LECs were stimulated with VEGF-A in the presence of either PD098059 or LY294002, inhibitors that selectively block MEK1 and PI3-K, respectively. Treatment of LECs with PD098059 (5 µM) completely blocked VEGF-A-mediated activation of ERK1/2 without affecting the phosphorylation of proteins upstream of MEK (Fig. 5A). Likewise, LY294002 (10 µM) specifically inhibited VEGF-A-induced phosphorylation of Akt but not ERK1/2 (Fig. 5B). The effect of PD098059 and LY294002 on VEGF-A-induced proliferation of LECs was then evaluated by the Cell Titer Blue assay. VEGF-A significantly induced LEC proliferation compared to the negative control (P<0.05) and was not affected by the addition of DMSO (vehicle for both PD098059 and LY294002) to the media (Fig. 5C,D). PD098059 (5 µM) and LY294002 (10 µM) significantly inhibited VEGF-A-induced proliferation of LECs (Fig. 5C,D). Transwell migration assays were then performed to determine the effect of PD098059 and LY294002 on VEGF-A-induced migration. VEGF-A significantly stimulated LEC migration (P<0.05) and was not affected by the presence of DMSO in the media. However, VEGF-A-induced migration was suppressed by PD098059 (5 µM) and LY294002 (10 µM, Fig. 5E,F).

## Discussion

Exhaustive investigation of the effect of VEGF-A on BECs has helped elucidate the signaling pathways regulating VEGF-A-induced hemangiogenesis. In contrast, the mechanisms underlying VEGF-A-induced lymphangiogenesis have not been widely examined and are poorly defined. The present study demonstrates for the first time that VEGF-A activation of VEGFR2 directly stimulates ERK1/2 and PI3-K/Akt mediated proliferation and migration of LECs. We propose that these cellular processes function together to drive VEGF-A-induced lymphangiogenesis.

To determine the role VEGF-A activation of VEGFR2 serves in lymphangiogenesis we used the monoclonal anti-VEGF-A antibody r84 which specifically blocks mouse and human VEGF-A activation of VEGFR2, but not VEGFR1. r84 significantly suppressed lymphangiogenesis *in vivo*, suggesting that blockade of VEGF-A activation of VEGFR2 is sufficient to inhibit lymphangiogenesis. However, it is unclear whether r84 directly inhibits lymphangiogenesis *in vivo* by preventing VEGF-A from activating VEGFR2 on LECs or indirectly suppresses lymphangiogenesis by affecting other cell types in the tumor microenvironment. Macrophages are reported to promote lymphangiogenesis and their recruitment to the tumor microenvironment is suppressed by r84 [29]. Additionally, fluid lost by leaky blood vessels in the tumor microenvironment may stimulate lymphangiogenesis. Anti-VEGF-A therapy reduces the permeability of blood vessels thereby silencing this potential trigger of lymphangiogenesis. Unfortunately, uncoupling VEGF-A's effect on multiple cell types is technically vexing. Future experiments using Cre/lox technology to specifically ablate Vegfr2 in LECs may help elucidate the extent to which this receptor directly promotes VEGF-A-induced lymphangiogenesis.

Although VEGF-A was reported to stimulate LEC proliferation and migration, it was previously unclear whether both VEGFR1 and VEGFR2 regulated these processes. We show that specific blockade of VEGF-A's interaction with VEGFR2 inhibits LEC proliferation and migration. Conversely, inhibition of VEGFR1 does not affect VEGF-A-induced proliferation or migration of LECs. To our knowledge, this is the first time the function of VEGFR1 has been examined in cultured LECs. These data reveal that VEGF-A activation of VEGFR2, not VEGFR1, directly promotes cellular processes required for lymphangiogenesis.

To identify the mechanisms by which VEGF-A activation of VEGFR2 stimulated LEC proliferation and migration, we analyzed VEGF-A-induced signaling pathways in LECs. We first focused on VEGF-A-induced auto-phosphorylation of VEGFR2. Tyrosines 951, 1054, 1059, 1175, and 1214 of VEGFR2 are phosphorylated following VEGF-A stimulation of BECs. We show for the first time that the same tyrosine residues become phosphorylated after treating LECs with VEGF-A. Of these tyrosines, Tyr 1175 may be the most important. Tyr 1175 is essential for proper VEGFR2 signaling in BECs. Knock-in mice in which Tyr 1173 (equivalent to Tyr 1175 in human VEGFR2) has been substituted to phenylalanine exhibit vascular defects similar to*Vegfr2* null mice [31]. Surprisingly, replacement of Tyr 1212 (equivalent to Tyr 1214 in human VEGFR2) with phenylalanine does not affect vascular development in mice [31]. Phosphorylation of Tyr 1175 leads to the recruitment and activation of PLCγ in BECs [32]. Subsequently PLCγ stimulates ERK1/2 activation via PKC [30]. The adaptor molecule Shb also binds to Tyr 1175 and is required for VEGF-A-induced activation of PI3-K signaling in BECs [33]. We show that VEGF-A/VEGFR2 activation in LECs stimulates PKC dependent phosphorylation of ERK1/2 and PI3-K dependent phosphorylation of Akt. These signaling events may be due to signaling initiated from Tyr 1175 of VEGFR2 in LECs.

The mutant phenotypes of several lines of genetically modified mice have recently implicated ERK1/2 as being an important signaling molecule in lymphangiogenesis. Mice expressing a constitutively active form of *Hras* exhibit lymphatic hyperplasia which is thought to be due to sustained activation of ERK1/2 [23]. Additionally, Spred1/2 double-knockout mice display hyperplastic lymphatics most likely because of dysregulation of ERK1/2 signaling [34]. Although these data suggest that ERK1/2 has a crucial function in the development of the lymphatic system, the precise role ERK1/2 serves in LECs was not previously defined. The phosphorylation of ERK1/2 is required for growth factor-induced proliferation of several cell types. We show that ERK1/2 phosphorylation is required for VEGF-A-induced proliferation of LECs. ERK1/2 can also influence cell migration by phosphorylating myosin light chain kinase [35]. We show that blockade of ERK1/2 phosphorylation inhibits VEGF-A-induced migration of LECs. Interestingly, inhibition of ERK1/2 activation does not block VEGF-A-induced migration of BECs [36]. This discrepancy may reflect an underlying difference between BECs and LECs.

PI3-K/Akt signaling is thought to be important in lymphangiogenesis. VEGFR3 promotes the survival of LECs by phosphorylating of Akt in a PI3-K dependent fashion [37]. Furthermore, *Akt1* mutant mice exhibit a hypoplastic network of lymphatic vessels [24]. We show that the phosphorylation of Akt is required for promoting VEGF-A/VEGFR2-induced viability and migration of LECs. This is most likely due to the activation of pro-survival pathways and endothelial nitric oxide synthase (eNOS) in LECs. In BECs, eNOS promotes VEGF-A-induced migration in a PI3-K/Akt dependent manner [38], [39]. PI3-K/Akt signaling also stimulates the phosphorylation of eNOS in LECs [38], [40]. Interestingly, guanylyl cyclase (GC), the only known NO receptor, is required for LEC migration [41]. These observations suggest a mechanism by which PI3-K/Akt signaling could regulate VEGF-A-induced migration of LECs.

In conclusion, we show for the first time that VEGF-A activation of VEGFR2, not VEGFR1, directly drives LEC proliferation and migration via the PI3-K and ERK1/2 signaling pathways. These data reveal that overlapping signaling pathways drive VEGF-A-induced cellular processes in BECs and LECs. Therefore, therapeutic agents targeting the VEGF-A/VEGFR2 axis could be useful to prevent the pathological formation of blood and lymphatic vessels.

## Materials and Methods

### Antibodies and reagents

The following commercially available antibodies were used: rabbit anti-phospho-ERK1/2 (Thr 202/Tyr 204, Cell Signaling #9101L); rabbit anti-ERK1/2 (Cell Signaling #9102); rabbit anti-phospho-PLC-γ (Tyr 783, Cell Signaling #2821S); rabbit anti-PLC-γ (Cell Signaling #2822); rabbit anti-phospho-VEGFR2 (Tyr 951, Cell Signaling #4991S; Tyr 1054 Upstate 04-894; Tyr 1059, Upstate 36-019; Tyr 1175, Cell Signaling #2478S; Tyr 1214, Upstate 07-374); rabbit anti-VEGFR2 (Cell Signaling #2479); rabbit anti-phospho-Akt (Ser 473, Cell Signaling #9271S); rabbit anti-Akt (Cell Signaling #9272); rabbit anti-LYVE-1 (abcam ab14917); and rabbit anti-Prox1 (abcam ab38692). r84 and XTL (control IgG) was received from Peregrine Pharmaceuticals Inc. Aurexis (control IgG) was generous gift from Dr. Phil Thorpe (UT Southwestern Medical Center). The VEGFR1 functional blocking antibody (6.12) was from ImClone. Rat anti-Flk2 was purified in our lab as previously describe [42] and used for immunofluorescence staining. PD098059 was purchased from Sigma-Aldrich (P215-5MG) whereas LY294002 was purchased from Calbiochem (440204). GF10203X was kindly provided by Dr. Alaksandra Basu (University of North Texas).

### Animal Experiments

Experiments performed with mice were performed in accordance with a protocol (APN 0974-07-05-1) approved by the IACUC of the University of Texas Southwestern Medical Center.

### Cell culture

Primary adult human dermal lymphatic endothelial cells (LECs) were purchased from LONZA (CC-2810). The certificate analysis sheet supplied by LONZA for each vial of cells indicated that greater than 95% of the cells were LECs (CD31 and podoplanin double-positive). This was determined by FACS. Cells were cultured on rat-tail collagen 1 (50 µg/ml) or 1% gelatin coated plastic ware in EGM-2MV media (LONZA CC-3125). Cells were not used past passage 6.

### Immunofluorescence staining of frozen sections

Frozen sections were fixed in acetone at −20°C and then briefly air-dried. PBS was used to dissolve OCT and then samples were blocked with 20% Aquablock (East Coast Biologics, PP82-P0691) in TBST. Primary antibody diluted in TBST+5% BSA was added and allowed to incubate overnight at 4°C. Slides were then washed with PBS+0.05% Tween20 and incubated for one hour with the appropriate secondary antibody (Jackson ImmunoResearch) diluted in TBST+5% BSA. Following another round of washes with PBS+0.05% Tween20, coverslips were mounted with ProLong Gold with DAPI (Invitrogen, P36931). Slides were analyzed using a Nikon Eclipse E600 microscope and images captured using NIS-Elements imaging software.

### Immunocytochemistry

LECs were cultured in 4-well chamber slides. Cells were then fixed with methanol, washed with PBS, permeabilized with PBS+0.1% TX-100, and then blocked with TBST+20% Aquablock. Anitbodies diluted TBST+5% BSA were added and allowed to incubate overnight at 4°C. Cells were then washed with PBS and incubated overnight with the appropriate secondary antibodies. Following another round of washes with PBS, coverslips were mounted with ProLong Gold with DAPI.

### Proliferation/Viability assays

LEC proliferation/viability was evaluated by the Cell Titer Blue assay (Promega G8081). This assay is based on the ability of living cells to convert the non-fluorescent compound resazurin to the fluorescent compound resorufin. LECs (3,000 cells per well) in EGM-2MV were seeded into the wells of a Falcon Optilux Black/Clear bottom 96-well plate. The next day, cells were serum-starved for 4 hours with OptiMEM reduced-serum media (Invitrogen 11058-021). During this time, recombinant human VEGF-A^165^ (R & D Systems 293-VE) was pre-incubated with r84, control IgG, or anti-VEGFR1 antibody for one hour. For the inhibitor experiments, LECs were treated with PD098059, LY294002, or DMSO while being serum starved. Next, EGM-2MV (positive control), OptiMEM (negative control), or recombinant human VEGF-A^165^ in the presence or absence of r84, anti-VEGFR1, control IgG, DMSO, PD098059 or LY294002 was added to the appropriate wells. After culturing cells for 48 hours at 37°C, 20 µl of Cell Titer Blue reagent was added to the wells and one hour later fluorescence was measured with a plate reader. The assay was run with 6 replicates for each experimental condition and performed at least twice.

### Migration assays

A modified Boyden chamber assay was performed to assess LEC migration. Cell culture inserts (8.0 µm pore size) were placed over wells of a 24-well tissue culture plate containing 500 µl of either EGM-2MV (positive control), OptiMEM (negative control), or recombinant human VEGF-A^165^ in the presence or absence of r84, control IgG, or anti-VEGFR1 antibody. Next, 200 µl of LECs (150,000 cells/ml) in OptiMEM reduced-serum media were seeded in the upper chamber of each insert and allowed to migrate overnight. For the inhibitor experiments, the indicated amounts of PD098059 and LY294002 were added to the upper and lower chambers. Cells that didn't migrate were removed from the upper chamber with a cotton swab. The membrane was then fixed and stained with the Diff-Quik stain kit (Dade Behring B4132-1A). The number of migrated cells was counted for 4 areas and values were normalized to the positive control. The assay was performed in triplicate and repeated twice.

### Western blot analysis

LECs were cultured on 6-well plates until near confluence, serum-starved overnight with OptiMEM reduced-serum, and then stimulated with recombinant human VEGF-A^165^ in the presence or absence of r84, control IgG, DMSO, PD098059 or LY294002. LEC were pre-treated with DMSO, PD098059, or LY294002 for one hour prior to stimulation. Following stimulation, cells were scraped in lysis buffer [mPER (Thermoscientific #78501) +Protease Inhibitor (Thermoscientific #78425) +Phosphatase Inhibitors I and II (Sigma-Aldrich P2850 and P5726)], spun for 10 minutes at 4°C, and then supernatants were transferred to new tubes. Equal amounts of total protein were separated by SDS-PAGE then transferred to PVDF membranes. Membranes were blocked for 30 minutes at room temperature with either TBST+5% BSA or TBST+5% non-fat milk, incubated overnight at 4°C with phospho-specific primary antibodies, washed with TBST, and then incubated for one hour at room temperature with the appropriate HRP-conjugated secondary antibodies. Bound antibodies were detected with the SuperSignal West Dura Extended Duration Substrate detection system (Thermoscientific #34076). Membranes were stripped then reprobed with antibodies to detect total levels of proteins.

### Statistical analysis

Data were analyzed using GraphPad Prism statistical analysis software (Version 5.0). All results are expressed as mean±SEM. Significance tested by unpaired student's T-test or ANOVA as indicated in the figure legends. Data were considered significant at *P*<0.05.

### Ethics Statement

Experiments performed with mice were performed in accordance with a protocol (APN 0974-07-05-1) approved by the IACUC of the University of Texas Southwestern Medical Center. Mice were housed in isolation cages located in a pathogen free facility in the NG building on the north campus of UT Southwestern. UT Southwestern has a letter of assurance on file with the Public Health Service, is registered as a research facility with the USDA, and is certified by the AAALAC. Our laboratory participates in voluntary inspections by IACUC and ARC staff at least twice per year. Mice were euthanized at the end of the proposed research or if they were deemed to be suffering. The method of euthanasia consisted of an inhalant overdose of carbon dioxide or isoflurane followed by cervical dislocation. These methods are consistent with the recommendations of the American Veterinary Medical Association (AVMA) Guidelines on Euthanasia.
